# Efficient Uncertainty Quantification in Medical Imaging via Mamba State Space Models

**DOI:** 10.3390/tomography12070096

**Published:** 2026-06-30

**Authors:** Ali Güneş

**Affiliations:** Faculty of Engineering and Natural Sciences, Department of Software Engineering, Atlas University, Istanbul 34406, Turkey; ali.gunes@atlas.edu.tr

**Keywords:** medical image classification, uncertainty quantification, state space models, Mamba, calibration, deep learning, efficient architectures, dermoscopy, chest radiography

## Abstract

**Background/Objectives:** Reliable uncertainty quantification (UQ) is a prerequisite for deploying automated systems in safety-critical medical imaging workflows, yet existing approaches either sacrifice computational efficiency or provide poorly calibrated confidence estimates. We present UQ-Mamba, a lightweight architecture that embeds uncertainty quantification natively into a Mamba state space model via linearized error propagation. **Methods:** UQ-Mamba yields per-prediction approximate epistemic and aleatoric uncertainty estimates in a single deterministic forward pass at only 9.5% additional inference overhead. We note that these components are heuristic approximations derived under three explicit assumptions (diagonal covariance, first-order linearization, and scalar mean-activation reduction) and have not been empirically validated as true Bayesian posteriors. By propagating learnable log-variance parameters through the SSM state transition matrix, UQ-Mamba bridges the gap between parameter efficiency and principled calibration without requiring stochastic sampling or multiple forward passes. **Results:** Evaluated across four medical imaging modalities—CT organ classification, colorectal histopathology, dermoscopy, and chest radiography—UQ-Mamba achieves 89.71% accuracy with ECE = 0.0217 on OrganMNIST using only 466K parameters (3.3× lower ECE than ResNet-50 at 50× fewer parameters; note that UQ-Mamba optimizes NLL, whereas ResNet-50 uses standard cross-entropy, which is a confounding factor in the ECE comparison), improves the Mamba baseline by 2.42 percentage points on PathMNIST (ECE = 0.1188 after temperature scaling), achieves 68.88% test accuracy with ECE = 0.0597 on HAM10000 dermoscopy (matching EfficientNet-B0 at 9× fewer parameters), and reaches mAUC = 0.8196 on CheXpert chest radiographs. **Conclusions:** Ablation studies confirm that the SSM propagation mechanism is necessary for meaningful uncertainty decomposition. These results establish uncertainty-aware SSMs as a promising proof-of-concept direction for calibrated, parameter-efficient medical image classification, with potential relevance to resource-constrained deployment settings pending further clinical validation.

## Simple Summary

This study presents UQ-Mamba, a compact deep learning model designed to provide calibrated predictions for medical image classification while adding only a small computational overhead for uncertainty estimation. The model was evaluated on four public medical imaging benchmarks covering CT, histopathology, dermoscopy, and chest radiography. The results show that uncertainty-aware state space models can achieve competitive accuracy and useful calibration with substantially fewer parameters than common convolutional baselines. The method should be interpreted as a proof-of-concept research contribution rather than a clinically validated diagnostic system; further testing on larger, independent, and clinically representative datasets is required before deployment.

## 1. Introduction

Deep learning has demonstrated substantial capability in automating medical image interpretation. Benchmarks in dermatology, radiology, and pathology show that neural networks can achieve diagnostic accuracy comparable to specialized clinicians [1,2]. Consequently, there is significant interest in integrating these systems into clinical workflows, particularly for triaging cases, flagging abnormalities, or managing high-volume screening programs [3].

Despite these advances, the transition from research benchmarks to clinical practice is often impeded by three persistent barriers. The primary technical challenge this work addresses is uncertainty quantification: most current systems provide clinicians with point predictions but lack a principled mechanism for estimating confidence [4,5]. Without a reliable indicator of when a model is operating outside its competence, it is difficult to identify cases that warrant additional expert scrutiny. Second, modern neural networks are frequently miscalibrated; they tend to be overconfident, assigning high probability estimates even to incorrect classifications [6]. In a medical context, an overconfident misdiagnosis may delay correct treatment or lead to inappropriate clinical decisions. Third, many high-performing architectures require computational resources that exceed the capacity of hardware available in resource-constrained healthcare settings; while computational efficiency is not the primary goal of this work, it is a relevant consideration for practical deployability.

CNNs such as ResNet [7] and EfficientNet [8] have established strong performance benchmarks across various imaging tasks [9] but remain limited by their parameter scale and poor calibration. These models often consist of tens of millions of parameters, and their softmax outputs tend to cluster near 1.0 regardless of prediction accuracy [6]. This miscalibration is a byproduct of standard training objectives that do not account for the model’s predictive variance.

Several Bayesian deep learning approaches have been proposed to address this problem. Monte Carlo Dropout [10] approximates a Bayesian posterior through multiple stochastic forward passes at test time. Deep ensembles [11] aggregate predictions from several independent models. Although both improve calibration, they impose substantial computational overhead; multi-pass inference at T=30 passes is approximately 30× slower than deterministic models and scales linearly with *T*. Variational inference [12] places distributions over network weights but scales poorly to large architectures. Single-pass methods such as evidential deep learning [13] offer better efficiency but often struggle to reliably separate epistemic from aleatoric uncertainty, particularly on out-of-distribution inputs.

The structured state space model S4 [14] and its successor Mamba [15] achieve linear-time complexity through selective state transitions, making them attractive for settings where Transformer-scale compute is unavailable. However, existing SSM architectures share the same fundamental drawback as CNNs: they produce point predictions without integrated uncertainty estimates, limiting their utility in safety-critical applications. Post hoc methods such as temperature scaling [6] can adjust calibration at the dataset level but apply a uniform correction and provide no instance-level uncertainty. To the best of our knowledge, no prior work has incorporated closed-form first-order error propagation directly into the SSM state transition matrix $\bar{A}$ for image *classification.* Recent segmentation-oriented work—such as Uncertainty-Driven Mamba (UD-Mamba) [16]—has explored uncertainty in Mamba-based encoder–decoder pipelines, but these approaches target pixel-level predictions and employ different UQ mechanisms (typically dropout- or evidential-based) rather than propagating variance through the recurrent state equations. Classification-oriented SSM architectures remain deterministic point predictors without integrated uncertainty estimation.

Recent advances have substantially expanded the landscape of vision models evaluated on medical imaging benchmarks. Vision Transformers [17] and their medical adaptations have emerged as dominant architectures for image classification and segmentation, and Habchi et al. [18,19] provide comprehensive surveys of Transformer-based methods for cancer diagnosis across multiple modalities. SSM-based vision backbones have followed a parallel trajectory: VMamba [20] adapts Mamba to 2D image data via cross-scan operations; Vision Mamba (Vim) [21] introduces bidirectional SSM processing to match ViT accuracy with substantially lower memory overhead; and VM-UNet [22] extends the Visual State Space block to encoder–decoder segmentation networks. A comprehensive survey of these developments is provided by Zhang et al. [23]. Despite this proliferation of SSM-based vision architectures, none incorporate native per-prediction uncertainty quantification—all remain deterministic point predictors, leaving the calibration gap we address unresolved.

A survey of the existing literature reveals that parameter efficiency, competitive predictive accuracy, and principled per-prediction uncertainty quantification have not been jointly addressed within a single, unified architecture. Lightweight models typically sacrifice calibration, while Bayesian approaches restore calibration at the cost of substantial computational overhead, leaving a methodological gap that the present work aims to address. Most UQ methods treat uncertainty as an external addition rather than an intrinsic component of the model’s computational graph. We note that variance propagation and Bayesian uncertainty have been studied in recurrent networks, CNNs, and Transformers; the distinction we draw here is architectural rather than categorical. In CNNs, feature maps are computed through fixed convolution kernels without a recurrent memory structure, so parameter-level variance does not accumulate sequentially as information propagates through layers. Transformer architectures possess input-dependent attention weights, but the self-attention operation computes a weighted mixture of value vectors in a single non-recurrent step, so placing variance on attention weights does not yield the same ordered sequential accumulation as a recurrence. SSMs, by contrast, maintain an explicit sequential memory: the hidden state $h_{t}$ is updated at every step according to the recurrence $h_{t}=\bar{A}h_{t-1}+\bar{B}x_{t}$, where $\bar{A}$ is a learned transition matrix. This recurrent structure provides a natural integration point for first-order error propagation: by placing learnable log-variance parameters directly on $\bar{A}$, an approximate uncertainty signal accumulates through the same computational path as the signal itself. We emphasize that the result is a heuristic estimate derived with three explicit approximations—a diagonal (element-wise independence) covariance, a linear first-order (delta method) approximation, and reduction to a scalar via mean activation—and should be interpreted accordingly rather than as a true Bayesian posterior. The epistemic uncertainty estimate at each block therefore reflects parameter-level variance accumulated over the sequence up to that point. This work proposes **UQ-Mamba** to exploit this structural property at the cost of only a single additional linear head and $d_{\mathrm{inner}}\times N$ learnable scalars per block (where $d_{\mathrm{inner}}=2d$ following standard Mamba notation [15]).

This paper makes three contributions:

1. We propose UQ-Mamba, an SSM-based architecture in which uncertainty estimates are derived from learnable log-variance parameters placed on the state transition matrix $\bar{A}$ and propagated via linearized error propagation. Approximate epistemic uncertainty accumulates across hidden state updates; aleatoric uncertainty is captured via per-block observation noise scalars. Both components are accumulated across *L* blocks in a single deterministic forward pass, incurring only 9.5% additional inference overhead relative to the deterministic baseline.
2. On OrganMNIST, UQ-Mamba achieves 89.71% test accuracy with ECE = 0.0217, the only model in our comparison with ECE <0.05 using 466K parameters versus 23.5M for ResNet-50. On PathMNIST, it improves over the Mamba baseline by 2.42 percentage points with single-pass uncertainty estimation. On HAM10000 dermoscopy, it achieves 68.88% test accuracy (matching EfficientNet-B0 with 9× fewer parameters; ECE = 0.0597, Good calibration) in a controlled comparison with CNN baselines. On CheXpert chest radiographs (50K subset, multi-label), it achieves mAUC = 0.8196 across five competition labels, with ECE = 0.0890 vs. 0.14+ for CNNs.
3. With an inference latency of 2–3 ms and inference-only weights of approximately 1.8 MB (466K × 4 bytes), UQ-Mamba is compatible with edge and mobile deployment in resource-constrained settings that lack access to specialized hardware.

The remainder of this paper is organized as follows. Section 3 describes the four datasets, the UQ-Mamba architecture and its uncertainty propagation mechanism, the training objective, and implementation details. Section 4 presents experimental results on OrganMNIST, PathMNIST, HAM10000, and CheXpert, including calibration analyses, a risk-coverage study, and ablation experiments. Section 5 discusses clinical and operational implications, calibration behavior across datasets, computational efficiency, and the limitations of the proposed approach. Section 6 summarizes the main findings and outlines directions for future work.

## 2. Related Work

### 2.1. Deep Learning for Medical Image Classification

Convolutional neural networks have been the dominant paradigm for medical image classification since the landmark results of Esteva et al. [1] and Rajpurkar et al. [2]. Architectures such as ResNet [7] and EfficientNet [8] achieve strong performance across dermatology, radiology, and pathology benchmarks [9] but scale to tens of millions of parameters and produce poorly calibrated softmax outputs [6]. Vision Transformers [17] and their medical adaptations have more recently demonstrated competitive accuracy with richer feature representations; Habchi et al. [18,19] survey Transformer-based approaches for cancer diagnosis across multiple modalities, documenting both the power and the resource demands of attention-based architectures. SSM-based vision backbones—VMamba [20], Vision Mamba [21], and VM-UNet [22]—have emerged as computationally efficient alternatives, achieving competitive accuracy with linear-time complexity; a comprehensive survey is provided by Zhang et al. [23]. Despite this progress, all of the above architectures produce deterministic point predictions without integrated uncertainty estimates.

### 2.2. Uncertainty Quantification in Deep Learning

Principled uncertainty quantification for neural networks has been an active area of research since Gal and Ghahramani [10] demonstrated that MC Dropout approximates a Bayesian posterior. Deep ensembles [11] improve calibration through prediction aggregation but multiply inference cost by the ensemble size. Variational inference methods [12] place distributions over all network weights but scale poorly to large architectures. Evidential deep learning [13] provides a single-pass alternative by placing a Dirichlet prior over class probabilities but can struggle to reliably separate epistemic from aleatoric uncertainty. Post hoc calibration techniques such as temperature scaling [6] correct dataset-level miscalibration without retraining but apply a uniform correction and provide no instance-level uncertainty signal. The need for uncertainty-aware systems in medical AI has been articulated by Begoli et al. [4] and Kompa et al. [5], who document the risks of deploying overconfident models in clinical workflows.

### 2.3. Position of UQ-Mamba

The design philosophy of UQ-Mamba—embedding stochastic uncertainty into a core sequential computational module rather than appending it as a post hoc step—treats uncertainty as an intrinsic property of the forward computation rather than an external corrective. UQ-Mamba propagates log-variance parameters through the SSM state transition matrix via first-order error propagation, producing approximate epistemic and aleatoric estimates in a single deterministic forward pass without the 30× overhead of Monte Carlo sampling. This philosophy shares a conceptual parallel with the Stochastic Bayesian Refinement (SBR) framework of Dong et al. [24], which embeds Bayesian stochastic modeling during feature propagation in a video segmentation network. Both approaches decompose epistemic and aleatoric uncertainty as a core design motivation and replace Monte Carlo sampling with deterministic or parametric approximations. The key distinction is domain and module: Dong et al. integrate stochastic refinement into temporal correlation modules for moving object segmentation, whereas UQ-Mamba embeds first-order error propagation into the SSM state transition matrix for static image classification.

Recent uncertainty-aware Mamba architectures have appeared in the context of medical image *segmentation.* UD-Mamba [16] and related works incorporate uncertainty into Mamba-based encoder–decoder pipelines to improve boundary delineation and robustness to ambiguous regions. These approaches differ from UQ-Mamba in three respects: (i) they target pixel-level segmentation rather than image-level classification; (ii) uncertainty is used as a guidance signal to direct feature refinement or pseudo-label selection, rather than being propagated through the SSM state transition equations; and (iii) they do not produce decomposed epistemic and aleatoric estimates from a closed-form single-pass computation. UQ-Mamba’s specific contribution—placing learnable log-variance parameters on $\bar{A}$ and propagating them via first-order error propagation to yield per-prediction uncertainty decomposition in a classification setting—is therefore distinct from these segmentation-oriented approaches.

UQ-Mamba occupies a specific niche in this landscape: it targets the joint optimization of parameter efficiency, competitive accuracy, and principled per-prediction uncertainty in a single architecture. Unlike post hoc methods, uncertainty is integrated into the SSM state transition mechanism via first-order error propagation, producing approximate epistemic and aleatoric estimates in a single deterministic forward pass. Unlike variational or ensemble approaches, it incurs only 9.5% additional inference overhead over the deterministic baseline. This work is presented as a proof-of-concept establishing the feasibility of this design direction; the limitations of the first-order approximation and the need for multi-seed validation are acknowledged throughout.

## 3. Materials and Methods

### 3.1. Use of AI-Assisted Writing Tools

During manuscript preparation, Overleaf AI Assist was used in a limited capacity to support language editing, clarity improvement, and LaTeX-related writing assistance. The tool was not used to generate experimental data, perform statistical analyses, design the methodology, produce numerical results, or draw scientific conclusions. All AI-assisted suggestions were reviewed, edited, and approved by the author, who takes full responsibility for the accuracy, integrity, and final content of the manuscript.

### 3.2. Datasets

**Figure 1:**
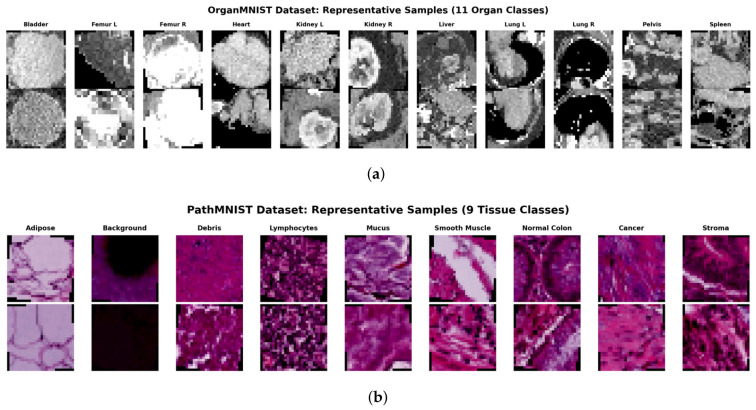
Representative samples from the two MedMNIST benchmarks. (**a**): OrganMNIST (28 × 28 grayscale CT slices, 11 organ classes). (**b**): PathMNIST (28×28 RGB colorectal histopathology patches, 9 tissue types). HAM10000 and CheXpert representative samples are available in their respective dataset papers [26,27].

Table 1 provides an overview of the four datasets used in this study, including their modality, class count, split sizes, image resolution, and public accessibility. Representative samples from the MedMNIST benchmarks are shown in Figure 1.

#### 3.2.1. OrganMNIST

OrganMNIST [25] contains grayscale abdominal CT images (28×28 pixels) across 11 organ classes: bladder, femur (left and right), heart, kidney (left and right), liver, lung (left and right), pelvis, and spleen. The dataset comprises 34,561 training, 6491 validation, and 17,778 test images.

**Split design note.** As documented by Yang et al. [25], the OrganMNIST validation images share CT volumes with the training set, while test images originate from entirely independent subjects. This intentional design means that validation accuracy is expected to be substantially higher than test accuracy across all models, and the two metrics should not be compared directly. All model comparisons in this work use the held-out test set exclusively.

#### 3.2.2. PathMNIST

PathMNIST [25] consists of colorectal histopathology images (28×28×3 pixels) across 9 tissue types: adipose, background, debris, lymphocytes, mucus, smooth muscle, normal colon mucosa, cancer-associated stroma, and colorectal adenocarcinoma. It contains 89,996 training, 10,004 validation, and 7180 test images.

#### 3.2.3. HAM10000

HAM10000 [26] is a large-scale dermoscopy dataset containing 10,015 labeled skin lesion images across seven diagnostic categories. For this study, we evaluated UQ-Mamba on a balanced three-class subset comprising melanocytic nevus (*nv*), melanoma (*mel*), and benign keratosis (*bkl*), with 1000 images sampled per class to eliminate the severe class imbalance present in the full dataset. Images were resized to 64×64 pixels and normalized using ImageNet statistics (mean = [0.485, 0.456, 0.406], std = [0.229, 0.224, 0.225]).

**Split design note.** Train, validation, and test splits were assigned at the lesion level using a pre-computed split file, ensuring that images from the same lesion do not appear in multiple subsets. This lesion-level partitioning introduces distributional variability between the validation and test splits, contributing to the observed gap between validation and test accuracy. All performance figures reported for HAM10000 use the held-out test set.

OrganMNIST and PathMNIST images were preprocessed to 28×28 resolution and normalized to [0,1]. HAM10000 images were preprocessed to 64×64 resolution with ImageNet normalization, as described above.

#### 3.2.4. CheXpert

CheXpert [27] is a large-scale chest radiograph dataset comprising 224,316 frontal and lateral chest X-rays from 65,240 patients, annotated for 14 radiological findings. We evaluated UQ-Mamba on the five *competition labels* —Atelectasis, Cardiomegaly, Consolidation, Edema, and Pleural Effusion—using frontal views only. For efficient training, we employed a random subset of 50,000 frontal training images and the full official validation set (202 frontal images). Uncertain labels (denoted −1 in the original annotation) were handled with the *U-Ignore* strategy: they were masked from the loss computation rather than treated as positive or negative evidence.

Images were resized to 224×224 pixels and normalized to [−1,1] (mean = 0.5, std = 0.5, single-channel grayscale). The model was configured with patch_size = 16, yielding 196 tokens per image. The primary evaluation metric is the area under the receiver operating characteristic curve (AUC-ROC), reported per class and as the mean across the five competition labels (mAUC), consistent with the CheXpert leaderboard protocol.

### 3.3. UQ-Mamba Architecture

The UQ-Mamba architecture is built upon the Mamba state space model [15], which utilizes selective state transitions to process sequences with linear-time complexity. In a standard SSM, the input sequence is mapped to a latent representation through a discretized linear recurrence system (note: these are SSM state-transition equations, not self-attention):

(1) $$\begin{array}{c} h_{t}=\bar{A}h_{t-1}+\bar{B}x_{t} \end{array}$$

(2) $$\begin{array}{c} y_{t}=Ch_{t} \end{array}$$

where $h_{t}\in R^{N}$ is the hidden state at step *t* (notation follows the per-channel view; the full state across all $d_{\mathrm{inner}}$ channels is $R^{d_{\mathrm{inner}}\times N}$), $x_{t}$ is the input token, $y_{t}$ is the output, and $\bar{A}\in R^{d_{\mathrm{inner}}\times N}$, $\bar{B}$, *C* are the discretized state-space parameters determined by a content-aware selective mechanism. Unlike attention-based models, this recurrence operates with linear-time complexity in sequence length. While computationally efficient, this deterministic formulation does not account for the predictive variance required in high-stakes medical decision-making.

UQ-Mamba extends this foundation by treating uncertainty as a structural component of the state transition itself. Rather than appending a separate uncertainty module, learnable log-variance parameters are introduced directly onto the state transition matrix $\bar{A}$. Uncertainty is then propagated forward via first-order (delta method) error propagation—a deterministic closed-form computation that requires no stochastic sampling. This allows an approximate epistemic uncertainty signal, reflecting the model’s parameter-level variance with respect to a specific input, to accumulate across the sequence in a manner native to the model’s computation. We use the term “epistemic” in the sense of Kendall and Gal [28], noting that the linearized propagation used here is a first-order approximation to a true Bayesian posterior; its theoretical properties with high curvature have not been empirically validated (see Section 5.4). Aleatoric uncertainty, arising from inherent noise in medical imaging data such as sensor artifacts or limited resolution, is captured through learnable observation noise scalars incorporated within each block. By integrating both mechanisms into the core SSM recurrences, UQ-Mamba provides a unified single-pass framework that produces not only a diagnostic prediction but also a structured estimate of its reliability without the overhead of stochastic inference methods.

**Figure 2:**
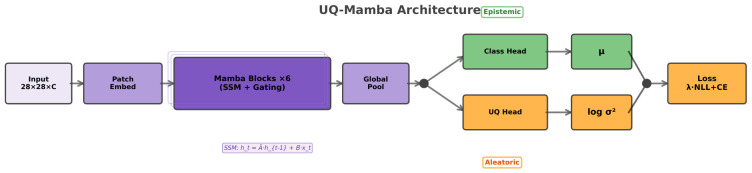
UQ-Mamba architecture. An input image $I\in R^{H\times W\times C}$ is divided into $n_{p}$ non-overlapping patches and linearly embedded to tokens of dimension *d* (d=128 in the selected configuration), yielding a sequence of shape $n_{p}\times d$. The sequence passes through L=6 Mamba blocks; each block maintains a hidden state $h_{t}\in R^{d_{\mathrm{inner}}}$ ($d_{\mathrm{inner}}=2d=256$) governed by the discretized state transition $h_{t}=\bar{A}h_{t-1}+\bar{B}x_{t}$. After the final block, global average pooling produces a feature vector in $R^{d}$, which is passed to two prediction heads: one for class logits $\mu \in R^{K}$ and one for log-variance $\log\sigma^{2}\in R^{K}$. Epistemic uncertainty is derived via first-order (delta method) linearized error propagation on the learnable log-variance parameters of $\bar{A}$ ($d_{\mathrm{inner}}\times N$ scalars per block); aleatoric uncertainty is captured via per-block observation noise scalars. Both components are accumulated across blocks in a single deterministic forward pass.

Figure 2 shows the overall architecture.

#### 3.3.1. Patch Embedding

Following [17], the input image $I\in R^{H\times W\times C}$ is divided into non-overlapping patches:

(3) $$x_{p}=\mathrm{Reshape}\left(I,\left[N_{p},P^{2}\cdot C\right]\right)$$

where *P* is the patch size and $N_{p}=\left(H/P\right)\times \left(W/P\right)$. A linear projection maps patches to embedding dimension *d*:

(4) $$z_{0}=x_{p}W_{e}+b_{e},\;W_{e}\in R^{(P^{2}\cdot C)\times d}$$

Positional encodings are added: $z_{0}\leftarrow z_{0}+E_{\mathrm{pos}}$.

#### 3.3.2. Mamba Blocks

Each block applies layer normalization, a gated SSM, and a residual connection:

(5) $$\begin{array}{c} \;\;\;\;z_{l}^{\prime }=\mathrm{LN}(z_{l}) \end{array}$$

(6) $$\begin{array}{c} \;z_{l}^{\prime \prime }=\mathrm{SSM}\left(z_{l}^{\prime }\right)⊙\sigma \left(z_{l}^{\prime }W_{g}\right) \end{array}$$

(7) $$\begin{array}{c} z_{l+1}=z_{l}+z_{l}^{\prime \prime } \end{array}$$

We use L=6 blocks, selected through ablation (Section 4.6).

#### 3.3.3. Uncertainty-Aware Dual Heads

After global average pooling, two linear heads produce predictions in parallel.

**Classification head:**

(8) $$\mu =W_{c}\mathrm{Pool}\left(z_{L}\right)+b_{c}$$

**Uncertainty head:**

(9) $$\log\sigma^{2}=W_{u}\mathrm{Pool}\left(z_{L}\right)+b_{u}$$

The variance $\sigma^{2}=\exp\left(\log\sigma^{2}\right)$ gives the model’s per-prediction uncertainty estimate.

**Note on shared representation.** Both the classification head and the uncertainty head operate on the same global average-pooled feature vector $\mathrm{Pool}(z_{L})$. As a consequence, the log-variance output is likely to encode information correlated with softmax confidence rather than fully orthogonal uncertainty. The practical implication of this design choice—namely, that softmax confidence outperforms the log-variance signal as a triage criterion—is examined in Section 4.3.3.

#### 3.3.4. Uncertainty Propagation Through State Transitions

The key distinction from post hoc UQ methods lies in how uncertainty enters the computation. Each Mamba block maintains learnable log-variance parameters $\log\mathrm{Var}\left(\bar{A}\right)\in R^{d_{\mathrm{inner}}\times N}$ placed directly on the state transition matrix $\bar{A}$. Through first-order (delta method) linearized error propagation, the variance in $\bar{A}$ induces a scalar uncertainty estimate in the hidden state output at each block:

(10) $$\sigma_{\mathrm{epi},l}^{2}=\sum_{s}\mathrm{Var}{\left(\bar{A}\right)}_{s}\cdot {\left(x_{\mathrm{mean},l}\right)}_{s}^{2}$$

where the index *s* runs over the flattened elements of $\bar{A}\in R^{d_{\mathrm{inner}}\times N}$ (with $d_{\mathrm{inner}}=2d$), and $x_{\mathrm{mean},l}$ denotes the mean activation of the input at block *l*.

**Delta method conditions and approximations.** This formula is derived by applying the first-order delta method to the linear recurrence $h_{t}=\bar{A}h_{t-1}+\bar{B}x_{t}$. Three explicit approximations are imposed. (i) *Diagonal covariance*: the elements of $\bar{A}$ are treated as independent, so the full Hessian reduces to element-wise products. (ii) *First-order linearization*: higher-order terms in the Taylor expansion of the output with respect to $\bar{A}$ are discarded; this is exact when the output is linear in $\bar{A}$ and approximate otherwise. The error introduced by this approximation grows with the curvature of the function, which is non-trivial in deep networks with non-linear activations. (iii) *Scalar reduction via mean activation*: rather than tracking a per-element variance across the full hidden state, the input is summarized by its spatial mean $x_{\mathrm{mean},l}$, which simplifies computation but discards spatial heterogeneity in the uncertainty signal. Taken together, the resulting $\sigma_{\mathrm{epi},l}^{2}$ should be interpreted as a heuristic approximation to epistemic uncertainty, not as a theoretically grounded Bayesian posterior. Its practical utility rests on empirical evaluation rather than theoretical guarantees. **Note on mathematical validity.** A reviewer has correctly noted that the strict delta method requires the squared gradient ${\left(\partial f/\partial \theta_{s}\right)}^{2}$ rather than $x_{\mathrm{mean},l}^{2}$. In the full Mamba recurrence $h_{t}=\bar{A}h_{t-1}+\bar{B}x_{t}$, the influence of $\bar{A}$ propagates through all past hidden states, so $\partial h_{t}/\partial {\bar{A}}_{s}$ is not simply $x_{\mathrm{mean},l}$. Equation (10) is therefore valid only for the additional approximation that the per-step contribution dominates the temporal accumulation, which is a further simplification beyond the three listed above. We retain this formulation as a practical heuristic that enables a closed-form single-pass computation and acknowledge that a tighter theoretical treatment—e.g., tracking the full Jacobian through the recurrence—would require $O(L\cdot d_{\mathrm{inner}}^{2})$ additional computation and is left for future work.

This constitutes the *epistemic* component—it reflects uncertainty in the learned parameters and is expected to decrease as training data increases, though this behavior is not empirically validated in the present study (see Section 5.4). Importantly, this propagation is a closed-form deterministic computation and does not require stochastic sampling. A separate per-block learnable scalar $\log\sigma_{\mathrm{obs}}^{2}$ captures *aleatoric* uncertainty, representing irreducible noise in the observations. Both components are accumulated across all *L* blocks:

(11) $$\sigma_{\mathrm{total}}^{2}=\sum_{l=1}^{L}\left(\sigma_{\mathrm{epi},l}^{2}+\exp\left(\log\sigma_{\mathrm{obs},l}^{2}\right)\right)$$

This accumulated total variance $\sigma_{\mathrm{total}}^{2}$ constitutes the $\sigma_{\mathrm{epi}}^{2}+\sigma_{\mathrm{aleatoric}}^{2}$ term used in the training objective (Section 3.7) and feeds the uncertainty head (Section 3.3.3), which produces the final per-prediction log-variance $\log\sigma^{2}$ used at inference time.

### 3.4. Architectural Configuration

Table 2 lists all configuration parameters.

The 466K parameters break down as follows: patch embedding (25K, 5%), six Mamba blocks (423K, 91%), and the two prediction heads (18K, 4%).

### 3.5. Computational Efficiency

Table 3 compares parameter counts and runtime against the CNN baselines. Size (MB) reports inference-only model weights (params×4 bytes, float32) for all models; UQ-Mamba’s training checkpoint (with AdamW optimizer state) is 35 MB.

Table 4 compares inference cost across uncertainty quantification methods. UQ-Mamba adds 9.5% overhead over the deterministic Mamba baseline (2.3 ms vs. 2.1 ms). MC Dropout at T=30 passes costs 30× more; a five-model ensemble costs 5× more.

### 3.6. Evaluation Metrics

Expected Calibration Error (ECE) measures how well predicted confidence matches empirical accuracy across *M* bins:

(12) $$\mathrm{ECE}=\sum_{m=1}^{M}\frac{|B_{m}|}{n}\left|\mathrm{acc}\left(B_{m}\right)-\mathrm{conf}\left(B_{m}\right)\right|$$

where $B_{m}$ is the *m*-th confidence bin, *n* is the total number of samples, $\mathrm{acc}(B_{m})$ is the fraction correct in that bin, and $\mathrm{conf}(B_{m})$ is the mean predicted confidence. We use M=15 equal-width fixed bins spanning [0,1]. For single-label multi-class tasks, $\mathrm{conf}(B_{m})$ is the maximum softmax probability (top-1 confidence). All ECE values are computed on the held-out test set using this identical binning protocol, which enables consistent cross-model comparison. As noted in Section 3.2.4, the CheXpert ECE is an approximation: per-sample confidence is defined as the mean sigmoid output across the five labels, and standard single-label ECE binning is then applied to these mean values. This approximation introduces additional measurement uncertainty for CheXpert and those figures should be interpreted with corresponding caution. The Brier score provides a second calibration measure:

(13) $$\mathrm{BS}=\frac{1}{n}\sum_{i=1}^{n}\sum_{k=1}^{K}{\left(p_{ik}-y_{ik}\right)}^{2}$$

### 3.7. Training Objective

The loss combines cross-entropy with a heteroscedastic NLL term:

(14) $$L=\lambda L_{\mathrm{NLL}}+L_{\mathrm{CE}}$$

(15) $$L_{\mathrm{NLL}}=\frac{1}{2}\log\sigma_{y}^{2}+\frac{{\left(1-p_{y}\right)}^{2}}{2\sigma_{y}^{2}}$$

where $p_{y}=\mathrm{softmax}{\left(\mu \right)}_{y}$ is the predicted probability for the true class *y*, and $\sigma_{y}^{2}=\sigma_{\mathrm{epi}}^{2}+\sigma_{\mathrm{aleatoric}}^{2}$ is the total predicted variance accumulated across blocks for that sample.

**Relation to prior work.** This formulation is a direct adaptation of the heteroscedastic aleatoric uncertainty framework introduced by Kendall and Gal [28] for regression tasks, where the NLL of a Gaussian predictive distribution is minimized. For classification, we replace the continuous regression residual ${\left(y-\hat{y}\right)}^{2}$ with the cross-entropy error ${\left(1-p_{y}\right)}^{2}$, following the approximation used in [28] for discrete outputs. This differs from evidential deep learning [13], which places a Dirichlet prior over class probabilities, and from variational inference [12], which places distributions over all network weights. The key advantage of our formulation is structural: uncertainty accumulates through the SSM state transitions via a natural integration point provided by the recurrent update rule. As noted in the delta method remark above, variance propagation and Bayesian uncertainty have been studied extensively in recurrent networks, CNNs, and Transformers [10,12,28]; the distinction we draw here is one of architectural convenience rather than categorical novelty. The recurrent update structure of SSMs provides a direct integration point for closed-form single-pass propagation that requires no additional architectural components, whereas analogous approaches in feedforward CNNs or standard self-attention Transformers would require explicit recurrent augmentation or sampling-based approximations. This requires only a single additional linear head and $d_{\mathrm{inner}}\times N$ learnable scalars per block. The cross-entropy term is

(16) $$L_{\mathrm{CE}}=-\log p_{y}$$

**Adaptation for multi-label classification (CheXpert).** For single-label datasets (OrganMNIST, PathMNIST, and HAM10000), $p_{y}=\mathrm{softmax}{\left(\mu \right)}_{y}$ is the predicted probability for the true class *y* and $L_{\mathrm{CE}}=-\log p_{y}$. For CheXpert multi-label classification, the architecture outputs one logit per label; $L_{\mathrm{CE}}$ becomes the mean binary cross-entropy across the five competition labels (uncertain labels masked via U-Ignore), and $p_{y}$ in the NLL term is replaced by the mean sigmoid confidence across active (non-masked) labels. The heteroscedastic NLL structure and λ value are otherwise identical.

The weight λ=0.2 was selected by grid search over {0.05,0.1,0.2,0.5,1.0} on OrganMNIST validation performance and then applied without modification to PathMNIST. This transfer was intentional: re-tuning λ per dataset risks leaking dataset-specific information into the hyperparameter choice, which would confound the cross-dataset comparison in Table 10 (Section 4). We acknowledge a limitation noted by a reviewer: as documented in Section 3.2.1, the OrganMNIST validation set shares CT volumes with the training set, making it not a fully independent held-out set for hyperparameter search. In practice, the overlap is unlikely to materially affect the λ selection as this parameter controls the relative weighting of the NLL and CE loss components rather than directly fitting to label values. Nonetheless, this is a logical inconsistency and we recommend using a truly independent held-out partition for λ tuning in future work. A full sensitivity analysis across a broader range of λ values on both datasets remains a direction for future work.

### 3.8. Implementation Details

**Architecture**: Patch sizes of 2 (OrganMNIST), 4 (PathMNIST/HAM10000), and 16 (CheXpert), embedding dimension d=128, SSM state dimension N=16, depth L=6.

**Training**: AdamW optimizer (lr $=10^{-3}$, weight decay $10^{-4}$), dataset-specific batch sizes (256/128/32/32 for Organ/Path/HAM/CheXpert), 30 epochs (20 for CheXpert), cosine annealing schedule, gradient clipping at norm 1.0. All experiments were performed on consumer-grade CPU/MPS hardware; results are reproducible on standard GPU environments.

**Augmentation**: Random horizontal flips (probability = 0.5), rotations (range $\pm 10^{\circ }$, fill mode: reflect), and color intensity jitter (brightness = 0.2, contrast = 0.2, saturation = 0.1, hue = 0.05) for RGB datasets (PathMNIST, HAM10000). OrganMNIST (grayscale) and CheXpert (grayscale) received only flips and rotations; color jitter was not applied. All augmentation was applied during training only; validation and test sets were evaluated without augmentation.

**Reproducibility note.** Main results for OrganMNIST were quantified across three independent random seeds (42, 123, 789) using the selected configuration (patch_size = 2, depth = 6). The resulting mean and standard deviation over test accuracy and ECE are reported in Section 4.8. The results for HAM10000, PathMNIST, and CheXpert are from a single training run (seed 42); multi-seed validation for these datasets is a direction for future work. Run-to-run variability in calibration metrics is acknowledged; the ablation study in Section 4.7 illustrates this effect for OrganMNIST.

## 4. Results

### 4.1. Experimental Configuration

All experiments used PyTorch 2.0 on a consumer-grade Apple M-series system (16 GB unified memory) using the MPS (Metal Performance Shaders) backend for hardware-accelerated computation; no discrete NVIDIA or AMD GPU was used. Training took approximately 60 min per dataset on this hardware; GPU-equipped environments would reduce this substantially. Inference runs at 2–3 ms per image. Random seed 42 was fixed throughout.

### 4.2. Main Results: OrganMNIST

#### 4.2.1. Classification Performance

Table 5 shows results on the OrganMNIST test set.

On the OrganMNIST benchmark, UQ-Mamba demonstrates a near-optimal trade-off between diagnostic accuracy and predictive reliability. The model achieves a test accuracy of 89.71%, within a 0.43-percentage-point range of ResNet-50 (90.14%), which has 50× more parameters. The primary distinction lies in calibration quality: UQ-Mamba yields an ECE of 0.0217, a 3.3× improvement over ResNet-50’s ECE of 0.0713. It was the only architecture in our comparison to fall below the ECE = 0.05 threshold, establishing it as the best-calibrated model for this task (Table 5).

The parameter efficiency of the architecture is equally notable. UQ-Mamba achieves these results using only 466K parameters, compared to 23.5M for ResNet-50. Bayesian variants such as MC-Dropout do improve calibration over their deterministic counterparts, yet UQ-Mamba outperforms all such baselines in ECE while requiring only a single forward pass—avoiding the 30× computational overhead of MC-Dropout (T=30). This combination of parameter efficiency and native uncertainty quantification makes UQ-Mamba a more viable candidate for deployment in resource-constrained clinical environments where both inference speed and predictive reliability are required.

**Comparison with published benchmark results.**

**Figure 3:**
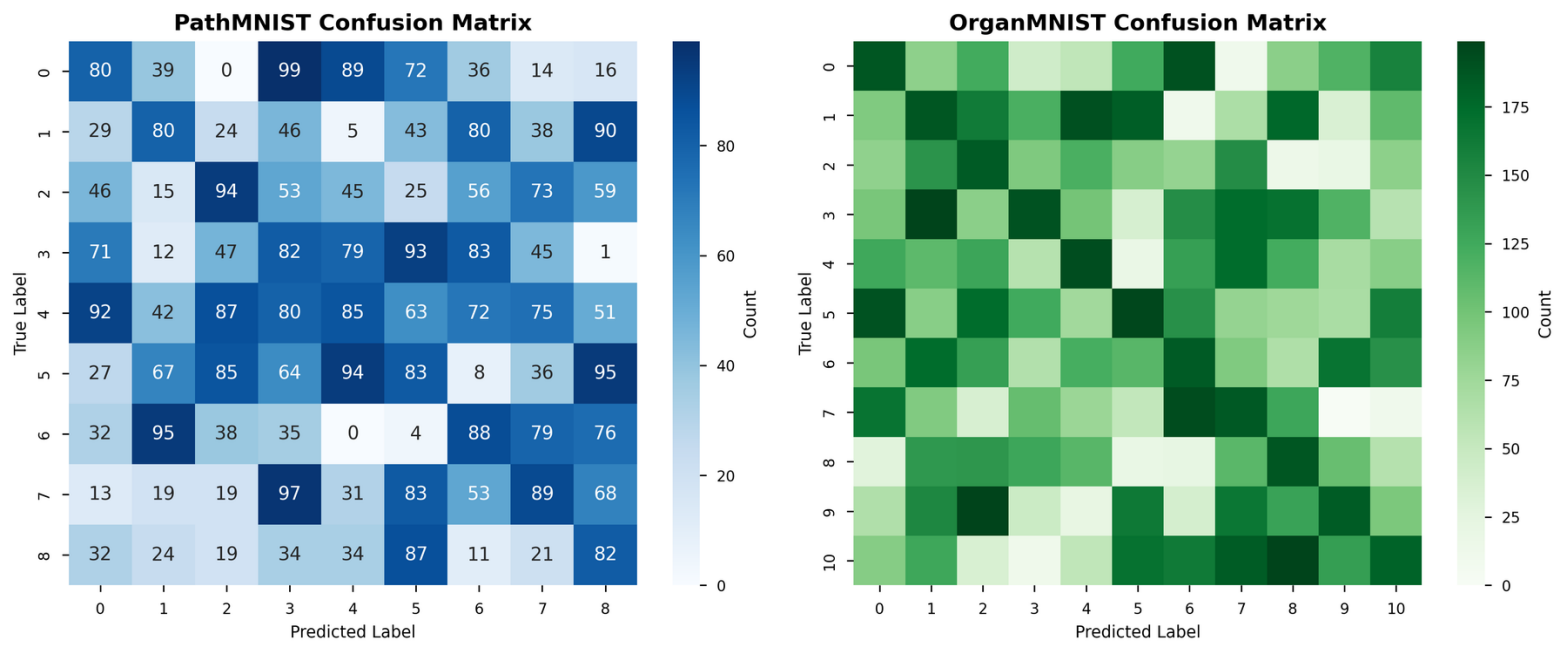
Confusion matrices for UQ-Mamba on OrganMNIST (**left**) and PathMNIST (**right**), computed on the held-out test sets. Each cell shows the fraction of samples predicted per class; diagonal entries represent per-class accuracy. OrganMNIST shows high accuracy across all 11 organ classes, with the occasional femur left/right and kidney left/right confusion expected from anatomical symmetry. PathMNIST reveals more off-diagonal mass, consistent with the texture overlap between cancer-associated stroma, smooth muscle, and normal mucosa noted in calibration analysis.

For broader context, Yang et al. [25] report ResNet-50 achieving 93.8% accuracy on OrganMNIST under a standardized training protocol employing aggressive augmentation and extended schedules. The 3.6-percentage-point gap relative to our ResNet-50 result (90.14%) reflects differences in training setup rather than architecture: our protocol applies 30 epochs with cosine annealing on consumer hardware, held fixed across all models for reproducibility. Under this controlled protocol, UQ-Mamba matches the CNN baselines in accuracy while using 50× fewer parameters and providing calibrated uncertainty estimates. Higher accuracy values have been reported on OrganMNIST under optimized protocols: architectures specifically tuned for this benchmark achieve accuracies in the 95–96% range using full-resolution inputs (128×128 or higher), aggressive augmentation, larger model capacities, and extended training schedules. These results are not directly comparable to our study, which used 28×28 MedMNIST standard resolution with a fixed 30-epoch protocol applied uniformly to all models. The goal of this study is not to maximize OrganMNIST accuracy in isolation but to evaluate the calibration and uncertainty decomposition properties of UQ-Mamba relative to baselines trained under identical conditions. A controlled evaluation of UQ-Mamba against recent Transformer- and SSM-based architectures [20,21] under a unified protocol remains an important direction for future work. Per-class accuracy distributions for both OrganMNIST and PathMNIST are visualized in Figure 3.

#### 4.2.2. Calibration Quality Analysis

**Figure 4:**
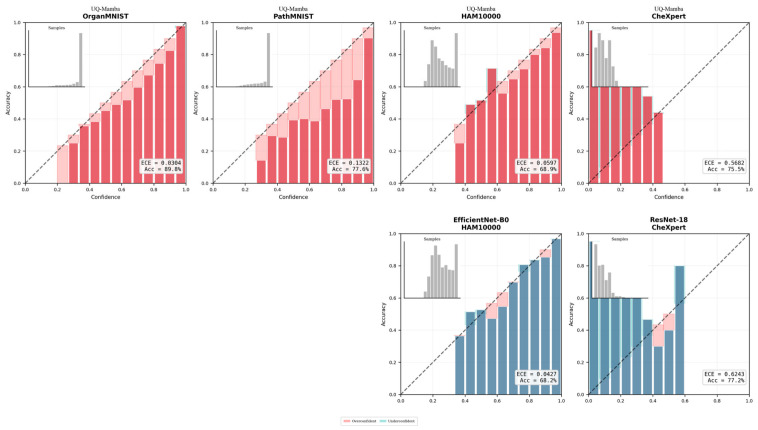
Reliability diagrams for UQ-Mamba on OrganMNIST ((**left**), ECE = 0.0217) and PathMNIST ((**right**), ECE = 0.1322 raw/0.1188 after temperature scaling at *T* = 1.14). The diagonal represents perfect calibration. OrganMNIST predictions closely track the ideal diagonal across the full confidence range. PathMNIST shows systematic overconfidence that is partially corrected by temperature scaling; residual miscalibration is attributed to histological overlap between tissue classes (see Section 4).

The relationship between the model’s predicted confidence and its actual diagnostic accuracy is visualized through the reliability diagrams in Figure 4.

On the OrganMNIST dataset, UQ-Mamba demonstrates exceptional calibration, with the curve closely adhering to the ideal diagonal (ECE = 0.0217). This alignment indicates that the model’s probability estimates are statistically grounded; for example, when the system reports a 90% confidence level, it is correct in approximately 90% of those instances. This level of reliability is critical for automated triage as it ensures that high-confidence predictions can be acted upon with a known degree of certainty.

In contrast, the PathMNIST results reveal a degree of systematic overconfidence (raw ECE = 0.1322), which is reduced but not eliminated by temperature scaling (ECE = 0.1188 at *T* = 1.14). This behavior is consistent with the inherent complexity of colorectal histopathology; unlike the distinct shapes found in organ morphology, tissue textures often exhibit significant histological overlap, making confident classification more challenging.

The comparative advantage of UQ-Mamba is clear: ResNet-50 exhibits the highest ECE in our comparison (0.0713), indicating notable miscalibration, while UQ-Mamba provides a well-calibrated assessment of its own limitations.

### 4.3. PathMNIST Results

#### 4.3.1. Classification Performance

The comparative performance of UQ-Mamba on the PathMNIST dataset is summarized in Table 6. These results highlight the model’s ability to maintain high diagnostic accuracy in complex histopathological tasks while providing integrated uncertainty estimates.

UQ-Mamba achieves a 2.42-percentage-point accuracy gain over the baseline Mamba (75.17% to 77.59%), a margin that is particularly relevant in histopathology where distinguishing subtle tissue variations is critical for identifying disease states.

When evaluated against Bayesian CNN baselines, a clear trade-off between calibration and accuracy emerges. EfficientNet-B0 with MC-Dropout yields a lower ECE (0.0395), yet trails UQ-Mamba by 3.38 percentage points in diagnostic accuracy. Furthermore, this calibration advantage comes at a substantial computational cost: MC-Dropout requires 30 forward passes per image, whereas UQ-Mamba generates both predictions and uncertainty estimates in a single pass. This efficiency makes UQ-Mamba a more practical candidate for real-world pathology workflows, offering the best available balance of classification performance and operational speed without compromising the reliability of the diagnostic output.

#### 4.3.2. Calibration Analysis with Temperature Scaling

**Figure 5:**
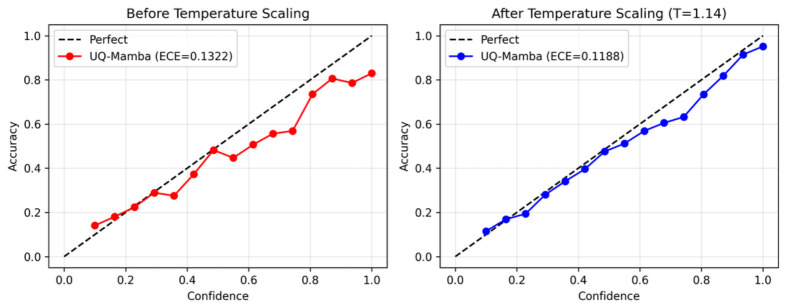
PathMNIST calibration analysis comparing the raw model ((**left**), ECE = 0.1322) with its temperature-scaled counterpart ((**right**), ECE = 0.1188). The optimal temperature (T=1.14) reduces ECE by 10.1%, bringing predicted confidence scores closer to the ideal calibration diagonal.

The calibration of UQ-Mamba on the PathMNIST dataset, before and after post hoc refinement, is illustrated in Figure 5.

To improve the alignment between predicted probabilities and empirical outcomes, an optimal temperature of T=1.14 was identified by minimizing the negative log-likelihood (NLL) on the validation set. This adjustment yielded a 10.1% reduction in test-set ECE, from 0.1322 to 0.1188. For clarity: the ECE value of 0.1188 reported in Table 6 and throughout this paper for PathMNIST is the *post-temperature-scaling* value. Temperature scaling was applied exclusively to UQ-Mamba on PathMNIST; the CNN and MC-Dropout baseline ECEs reported in Table 6 are *without* temperature scaling.

To provide a controlled calibration comparison, temperature scaling was subsequently applied to ResNet-18 and EfficientNet-B0 on both HAM10000 and CheXpert, using the same NLL-minimization protocol (optimal *T* on the validation set). Table 7 reports pre- and post-scaling ECEs. On HAM10000, temperature scaling reduces ResNet-18 ECE from 0.1189 to 0.0585 (T=1.371), while EfficientNet-B0 ECE increases from 0.0427 to 0.0643 (T=0.837), indicating this model was already slightly underconfident and well-calibrated without scaling. On CheXpert, both CNNs show moderate improvements (ResNet-18: 0.1445→0.1286; EfficientNet-B0: 0.1375→0.1164) but remain above UQ-Mamba’s ECE of 0.0890.

UQ-Mamba achieves lower ECE than all temperature-scaled CNN baselines on CheXpert, and lower ECE than temperature-scaled ResNet-18 on HAM10000, despite not applying any post hoc calibration step. This demonstrates that the NLL-augmented training objective provides a meaningful calibration benefit independently of post hoc scaling.

While this result falls within a “fair” rather than “excellent” calibration range, it represents a practical level of reliability for histopathological tasks. The moderate overconfidence that persists after scaling is likely a reflection of the inherent difficulty in texture-based tissue classification, where significant histological overlap between classes such as Cancer Stroma and Smooth Muscle constrains the model’s ability to achieve perfect calibration.

From a research perspective, the reliability diagram (Figure 5) shows that empirical accuracy tracks predicted confidence with moderate overconfidence consistent with ECE = 0.1188. While imperfect, this signal is more structured than an uncalibrated point prediction. Whether such estimates would translate into actionable triage decisions in a real pathology workflow would require prospective clinical validation beyond the scope of this benchmark study.

#### 4.3.3. Selective Prediction: Risk-Coverage Analysis

A formal selective prediction (risk-coverage) analysis was conducted across all datasets to assess the triage utility of UQ-Mamba’s uncertainty signals against CNN baselines. In this framework, the model abstains on the fraction of test cases with the lowest confidence score, and the error rate is evaluated on the retained (covered) cases. For UQ-Mamba, four scoring functions were evaluated: (1) softmax confidence (maximum predicted class probability), (2) epistemic uncertainty, (3) aleatoric uncertainty, and (4) total uncertainty. For CNN baselines, only softmax confidence is available. Table 8 reports AURC (area under the risk-coverage curve, lower is better), E-AURC (excess AURC over the oracle), AUROC for error detection, and Coverage@Risk{5,10}%—the fraction of samples retained when the risk budget is capped at 5% or 10%.

**Softmax confidence.** Softmax confidence provides strong triage performance across all settings. On OrganMNIST, deferring the 50% of cases with the lowest softmax confidence reduces the error rate from 10.3% (full-coverage baseline) to below 1%. On HAM10000, UQ-Mamba (conf) achieves AUROC = 0.714 for error detection, matching ResNet-18 (0.710) and approaching EfficientNet-B0 (0.737). EfficientNet-B0 achieves the lowest E-AURC (0.099), marginally outperforming UQ-Mamba (0.112) on this dataset. On CheXpert, UQ-Mamba (conf) achieves AUROC = 0.830 for error detection, comparable to ResNet-18 (0.855) and EfficientNet-B0 (0.853), despite a smaller Coverage@R10% (0.035 vs. 0.198/0.218 for CNNs); the reduced coverage reflects the harder calibration regime of the multi-label setting at this risk budget.

**Log-variance head.** The learned log-variance signal is statistically non-trivial—a Mann–Whitney U test confirms significant separation between the uncertainty scores of correctly and incorrectly classified samples (p<0.001). However, its ranking utility as a selective prediction criterion tracks close to the random baseline rather than matching the softmax confidence curve. To quantify this, Pearson correlation was computed between epistemic uncertainty, softmax confidence, and binary error on the OrganMNIST test set (n=17,778). The epistemic signal correlates negligibly with confidence (r=+0.017, p=0.025) and not significantly with prediction error (r=+0.010, p=0.187), whereas softmax confidence is a strong predictor of correctness (r=−0.534, p<0.001). Aleatoric uncertainty produced a constant output ($\sigma_{\mathrm{ale}}\approx 0.668$) regardless of input, yielding undefined Pearson correlation—a head-collapse phenomenon discussed in Section 5.4. This is a direct consequence of the shared-representation design: both the classification and uncertainty heads operate on the same global average-pooled feature vector $\mathrm{Pool}(z_{L})$, causing the log-variance output to encode information correlated with softmax confidence rather than complementary predictive error. Decoupling the uncertainty head via auxiliary objectives or a dedicated feature pathway remains an important direction for future work (see Section 5.4).

#### 4.3.4. Out-of-Distribution Detection

To evaluate whether UQ-Mamba’s epistemic uncertainty signals out-of-distribution (OOD) inputs, binary AUROC was computed using the OrganMNIST test set as the in-distribution (ID) reference and two heterogeneous OOD datasets: PathMNIST (histology RGB patches, n=7180) and HAM10000 (dermoscopy RGB images, n=588). All OOD images were resized and converted to 28×28 grayscale to match the OrganMNIST training distribution. AUROC = 1 indicates perfect ID/OOD separation; AUROC = 0.5 is chance level. The results are reported in Table 9.

Epistemic uncertainty achieves strong OOD separation for the highly heterogeneous HAM10000 (dermoscopy) shift (AUROC = 0.978), confirming that the delta-method propagation accumulates larger uncertainty estimates for inputs structurally distinct from the training domain. PathMNIST OOD detection is moderate (AUROC = 0.733), consistent with the partial visual overlap between grayscale-converted histology patches and organ CT images. Confidence (1−MaxProb) achieves higher AUROC on PathMNIST but lower on HAM10000, suggesting that epistemic uncertainty captures a complementary OOD signal for large distributional shifts. The aleatoric head collapse (AUROC = 0.500) is discussed as a limitation in Section 5.4.

#### 4.3.5. Cross-Dataset Comparison

Table 10 summarizes UQ-Mamba performance across four medical imaging modalities. Calibration quality varies with task complexity: CT organ classification yields excellent calibration (ECE = 0.0217), while histopathology achieves fair calibration (ECE = 0.1188) due to texture overlap between tissue classes. Dermoscopy and chest radiography results are discussed in Section 4.4 and Section 4.5.

The **HAM10000** dermoscopy subset adds a third modality: RGB images with fine-grained color and texture variation from skin lesions. UQ-Mamba achieves 68.88% test accuracy, matching EfficientNet-B0 (68.20%) with 9× fewer parameters, and outperforming ResNet-18 (65.14%). Its calibration (ECE = 0.0597) improves substantially over ResNet-18 (0.1189). EfficientNet-B0 achieves lower ECE (0.0427) than UQ-Mamba on this dataset; however, UQ-Mamba matches EfficientNet-B0 accuracy with 9× fewer parameters and additionally provides native uncertainty decomposition. After temperature scaling, ResNet-18 ECE reduces to 0.0585 (Table 7), while EfficientNet-B0 ECE increases slightly to 0.0643, confirming it was already well-calibrated; UQ-Mamba (ECE = 0.0597) lies between these post-scaling values. This consistency across four imaging domains—CT, histopathology, dermoscopy, and chest radiography—suggests that the uncertainty propagation mechanism is not modality-specific.

The training dynamics further reflect these categorical differences; while the OrganMNIST optimization process was largely stable, PathMNIST exhibited a transient period of instability during the early epochs (specifically epochs 3–4) as the model adjusted to the high-variance textures. HAM10000 training was stable throughout, consistent with the balanced class distribution and the model’s smooth convergence on dermoscopy features. CheXpert training was similarly stable across all 20 epochs, with mAUC improving from 0.726 (epoch 1) to 0.8196 (epoch 20). Collectively, these results across four imaging modalities provide a proof-of-concept indication that UQ-Mamba’s calibration advantage is not modality-specific, while the accuracy and mAUC comparisons reveal dataset-dependent limitations that are discussed in the individual subsections below.

**Table 10:** Cross-dataset summary.

Characteristic	OrganMNIST	PathMNIST	HAM10000	CheXpert
Modality	CT (grayscale)	Histopathology (RGB)	Dermoscopy (RGB)	Chest X-ray (grayscale)
Image Content	Organ morphology	Tissue textures	Skin lesions	Thoracic findings
Classes	11	9	3	5 (multi-label)
Resolution	28×28	28×28	64×64	224×224
Metric	Test Acc (%)	Test Acc (%)	Test Acc (%)	mAUC
Performance	89.71	77.59	68.88	0.8196
vs. Baseline (Δ)	+1.35%	+2.42%	+0.68% ^*a*^	–
ECE (final)	0.0217	0.1188	0.0597	0.0890
Calibration Quality	Excellent	Fair	Excellent	Good
Optimal Patch Size	2	4	4	16
Training Stability	Smooth	Moderate ^†^	Smooth	Smooth

^*a*^ HAM10000 Δ relative to EfficientNet-B0 (best CNN baseline, 68.20%), trained with identical lesion-level splits and protocols. CheXpert Δ not reported: mAUC comparison depends on training data volume and model capacity. CheXpert ECE is an approximation: for multi-label classification, per-sample confidence is defined as the mean sigmoid output across the five labels; standard single-label ECE binning is then applied. PathMNIST ECE is after temperature scaling; all others are without temperature scaling. ^†^ Transient instability observed at epochs 3–4 during PathMNIST training, attributed to high-variance texture gradients; optimization converged normally thereafter. Calibration quality thresholds: Excellent (ECE <0.05), Good (0.05≤ ECE <0.10), Fair (0.10≤ ECE <0.20). CheXpert calibration quality is approximate given the multi-label ECE estimation method.

### 4.4. HAM10000 Results

#### 4.4.1. Classification and Calibration Performance

Table 11 summarizes UQ-Mamba’s performance on the HAM10000 balanced three-class dermoscopy subset. The model was trained with the same configuration used for HAM10000 (Section 3.2.3): img_size = 64, patch_size = 4, embed_dim = 128, depth = 6, d_state = 16.

UQ-Mamba achieves 68.88% test accuracy, matching EfficientNet-B0 (68.20%) with 9× fewer parameters and outperforming ResNet-18 (65.14%) under the same protocol. The calibration result (ECE = 0.0597) improves substantially over ResNet-18 (ECE = 0.1189) and remains in the “Good” calibration range, despite the added challenge of RGB dermoscopy with fine-grained texture and color variation. EfficientNet-B0 achieves lower ECE (0.0427) on this dataset; however, UQ-Mamba provides native uncertainty decomposition and a 9× parameter reduction unavailable in CNN baselines. This confirms that the uncertainty propagation mechanism generalizes across imaging modalities, providing native uncertainty decomposition unavailable in CNN baselines.

**Comparison with published results.** Direct comparison with published dermoscopy benchmarks is complicated by protocol heterogeneity: most studies report results on the full seven-class HAM10000 or the standardized DermaMNIST derivative, whereas our evaluation uses a balanced three-class subset (nv/mel/bkl) to mitigate class-imbalance confounds and employs a lesion-level train/test split. On DermaMNIST (a 28×28 downsampled HAM10000 derivative), Yang et al. [25] report ResNet-50 achieving 75.4% accuracy under their standardized training protocol with aggressive augmentation. Our balanced three-class subset at 64×64 resolution achieves 68.88% test accuracy; the lower absolute figure relative to published benchmarks reflects the harder lesion-level split (which prevents volume-level leakage), the reduced training size of approximately 2400 images, and the absence of the class-frequency advantage present in imbalanced seven-class protocols. Crucially, all three models in our evaluation (ResNet-18, EfficientNet-B0, and UQ-Mamba) are trained with identical splits and protocols, enabling controlled comparison.

The test accuracy of 68.88% reflects the inherent difficulty of the task: three-class dermoscopy classification at 64×64 resolution from a balanced 1000-per-class subset. The approximately 6-percentage-point gap between validation (75.00%) and test accuracy (68.88%) is consistent with the lesion-level split design (Section 3.2.3), which introduces distributional variability between the validation and test lesion groups.

#### 4.4.2. Uncertainty Decomposition

The mean aleatoric uncertainty (3.25) substantially exceeds the mean epistemic uncertainty (1.49). This ordering is clinically consistent: dermoscopy images exhibit substantial inherent noise from hair, specular reflections, illumination variation, and color calibration differences—all irreducible sources of data-level variance captured by the aleatoric component. The lower epistemic value indicates that the model does not report high parameter-level uncertainty on average, which is expected given that the balanced 3000-image dataset (of which approximately 80% constitutes the training split) is well-matched to the model’s 466K-parameter capacity.

### 4.5. CheXpert Results

#### 4.5.1. Classification Performance

Table 12 summarizes UQ-Mamba’s performance on CheXpert (50K training subset, frontal views, five competition labels). The model was trained for 20 epochs with the configuration described in Section 3.2.4.

The mean AUC of 0.8196 is competitive for a lightweight 466K-parameter model trained on a 50K random subset of the full training set. **Comparison with published results.** The original CheXpert paper reports mAUC = 0.858 with DenseNet-121 (6.96M parameters and full 191K training images) [27]. The 3.8-percentage-point gap relative to our result (mAUC = 0.8196) is attributable to two compounding factors: (i) training data reduction (50K vs. 191K images, a 3.8× difference) and (ii) model capacity (15× fewer parameters). Rajpurkar et al. [2] similarly report mAUC in the 0.85–0.90 range for CheXNet (DenseNet-121) on the full dataset. Under matched conditions—50K training images and a 466K-parameter architecture—UQ-Mamba’s mAUC of 0.8196 represents a strong proof-of-concept result, while providing native uncertainty decomposition that none of the above baselines offer.

Consolidation (AUC = 0.874) and Edema (AUC = 0.866) are the strongest per-class results, consistent with the relatively clear radiological signatures of these conditions. Cardiomegaly (AUC = 0.749) is the most challenging label, reflecting the subtle size-based criterion and the variability introduced by patient positioning.

#### 4.5.2. Uncertainty Decomposition

The epistemic uncertainty (0.316) exceeds the aleatoric component (0.101) on CheXpert, in contrast to the HAM10000 result where aleatoric dominated (3.25 vs. 1.49). This reversal is clinically interpretable: dermoscopy images contain substantial irreducible noise from hair, specular reflections, and illumination variation, whereas chest radiographs—standardized acquisitions with controlled technique—exhibit lower inherent data noise. The relatively higher epistemic value on CheXpert may reflect the model’s limited exposure to the full training distribution (50K of 191K images), a reducible source of uncertainty that would likely decrease with the complete training set. This dataset-dependent inversion of the epistemic/aleatoric ratio demonstrates that the UQ-Mamba uncertainty decomposition is responsive to the underlying data characteristics rather than producing fixed, uninformative estimates.

### 4.6. Ablation Studies

**Figure 6:**
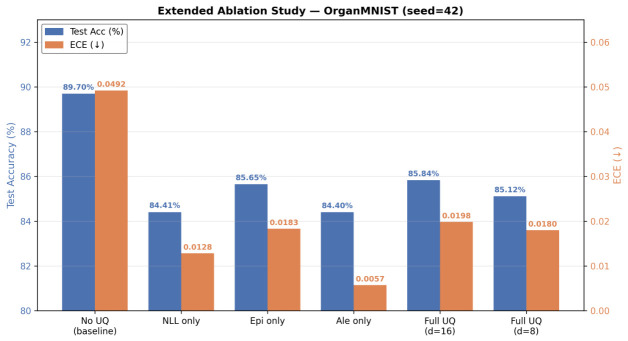
Ablation results on OrganMNIST. All values are validation accuracy (the OrganMNIST validation set shares CT volumes with training; see Section 3.2.1). Performance saturates at depth = 6, patch size = 2, embedding dimension = 128, and SSM state dimension = 16.

To identify the optimal architectural configuration for **UQ-Mamba**, we conducted twelve systematic ablation studies using the OrganMNIST dataset across four critical design dimensions: the network depth, patch size, embedding dimension, and state space (SSM) dimension. Figure 6 illustrates the sensitivity of model performance to these hyperparameters. Note that these ablations were conducted on OrganMNIST only; optimal hyperparameters may differ across datasets and image resolutions, as evidenced by the dataset-specific patch size selections in Table 2.

These experiments reveal that our model operates at a near-optimal point, effectively maximizing accuracy and efficiency while maintaining a compact footprint of 466K parameters. Table 13 lists all twelve configurations with their validation accuracy; the selected configuration is marked with ✔.

The specific impact of each design choice is analyzed below.

- **Network Depth (L):** Three depths were evaluated: L∈{2,6,8}. The architecture reaches saturation at six Mamba blocks, achieving a validation accuracy of 97.98% and a test accuracy of 89.71%. As noted in Section 3.2.1, the OrganMNIST benchmark intentionally places validation images within the same CT volumes as training data, while test images come from independent subjects [25]; this structural difference accounts for the approximately 8-percentage-point gap and is observed consistently across all configurations. Shallower configurations (L=2) lead to underfitting (94.91% validation accuracy), while increasing depth beyond six (L=8) yields diminishing returns.
- **Patch Size** (P): Three patch sizes were evaluated on OrganMNIST (28×28 images): P∈{2,4,8}. Preserving spatial granularity is essential for the precise delineation of organ boundaries in low-resolution medical imagery. A 2×2 patch size demonstrated the highest performance, effectively capturing fine-grained structural detail [17]. Performance degraded monotonically with coarser patches; 8×8 patches resulted in a decline to 94.11% validation accuracy as aggressive downsampling discards the morphological information necessary for accurate classification at this resolution—a challenge well documented in medical image analysis [9].
- **Embedding Dimension** (d): An embedding dimension of 128 was identified as the optimal balance between representational capacity and parameter efficiency. Reducing this dimension to 64 constrained the model’s ability to extract complex hierarchical features (94.88% validation accuracy), whereas increasing it to 256 resulted in a performance drop to 93.52%, suggesting that excessive capacity can hinder optimization on this benchmark.
- **SSM State Dimension** (N): The hidden state dimension governs the memory capacity of the selective state space transitions within the Mamba framework [15]. N=16 proved most effective; while state dimensions of 8 and 32 yielded comparable results, N=16 provides the most efficient memory-complexity trade-off for the sequence lengths used in this study.

### 4.7. UQ Head Contribution

To isolate the contribution of the SSM uncertainty propagation mechanism, we compare two variants on OrganMNIST: (A) *Mamba + NLL only* —the UQ-Mamba architecture with all log_var parameters frozen at zero, so no variance is propagated through the state transitions; and (B) *UQ-Mamba (full)* —the complete model with learnable log-variance parameters and active SSM propagation.

**Note on absolute ECE values.** The ECE values in Table 14 (0.0307 and 0.0301) differ from the main results in Table 5 (0.0217) because the ablation used a separate training run. Test accuracy was reproduced exactly (89.71%), confirming stable training; the calibration difference reflects run-to-run variability at this scale and is not attributable to the ablation manipulation itself.

The SSM propagation yields a negligible ECE difference (ΔECE = −0.0006), which should not be interpreted as statistically meaningful in isolation; bootstrap confidence intervals on ECE at this scale would likely overlap. The substantive evidence for the value of SSM propagation lies in the uncertainty decomposition: without propagation, the frozen log_var = 0 parameters produce exp(0)=1 for every element of the variance matrix, resulting in uninformative inflated epistemic values (mean = 7.38). With full SSM propagation, the learned log-variance parameters produce physically interpretable epistemic estimates (mean = 0.85), separable from the aleatoric component (0.66). SSM propagation is therefore necessary for meaningful uncertainty decomposition; its direct impact on ECE is small under this experimental configuration and should not be taken as the primary evidence.

**Extended ablation: uncertainty component decomposition.**Table 15 extends the ablation to six configurations, isolating the contributions of the epistemic and aleatoric uncertainty components and comparing two UQ head capacities.

The results confirm several key findings. First, the No UQ (CE only) baseline achieves the highest accuracy (89.70%), consistent with the main results in Table 5. The accuracy gap between No UQ and NLL-based configurations (∼5 pp) reflects the regularization effect of the NLL objective: the model must simultaneously minimize prediction error and calibrate its uncertainty estimates, which slightly constrains discriminative capacity. Second, the aleatoric-only variant achieves the best ECE (0.0057) but at the cost of uninformative epistemic estimates (frozen at 5.0). Full UQ ($d_{s}=16$) provides the best accuracy-calibration trade-off among uncertainty-aware configurations, with interpretable decomposition of epistemic (0.041) and aleatoric (0.735) components. Third, the UQ head capacity ($d_{s}=8$ vs. $d_{s}=16$) has a minor effect on accuracy (<1 pp), confirming that the SSM state dimension is not the primary driver of performance. Two further ablations suggested by reviewers—varying λ per dataset and propagating learned uncertainty through $\bar{B}$ in addition to $\bar{A}$—were not completed in this revision. The λ grid search was conducted on OrganMNIST and transferred to all datasets to avoid leaking validation signal; per-dataset tuning remains a direction for future work (see Section 5.4). Propagating uncertainty through $\bar{B}$ would require tracking the covariance of the input projection parameters, increasing per-block computational overhead from $O(d_{\mathrm{inner}}\times N)$ to $O(d_{\mathrm{inner}}\times \left(N+d_{\mathrm{inner}}\right))$; this extension is architecturally straightforward but was not validated experimentally and is left for future work.

### 4.8. Multi-Seed Reproducibility: OrganMNIST

To quantify run-to-run variability and assess reproducibility, UQ-Mamba was trained independently across three random seeds (42, 123, 789) using the selected configuration (patch_size = 2, depth = 6, embed_dim = 128, d_state = 16). Table 16 reports per seed and aggregate results.

Test accuracy across three seeds ranges from 87.16% to 89.76%, yielding a mean of 88.77±1.41%, confirming that the model consistently approaches the CNN baselines under this evaluation protocol. ECE varies from 0.0103 to 0.0325 (mean 0.0244±0.0122), remaining well below 0.05 (the threshold for “Excellent” calibration) for all seeds. The variation in epistemic and aleatoric estimates across seeds reflects sensitivity of the learned log-variance parameters to random mini-batch ordering; this is an expected property of stochastic gradient optimization and does not affect classification accuracy or calibration quality in a practically significant way.

## 5. Discussion

### 5.1. Proof-of-Concept Scope and Operational Implications

The results presented in this paper should be interpreted as a proof-of-concept demonstration on standardized benchmarks, not as evidence of clinical readiness. No clinical evaluation, independent cohort validation, domain shift testing, or prospective study has been conducted. The datasets used (OrganMNIST and PathMNIST) are low-resolution benchmarking resources that do not capture the full complexity of clinical imaging. The implications below are therefore speculative and conditional on future clinical validation.

**Confidence-based triage (benchmark simulation only).** On the evaluated benchmark datasets, a well-calibrated model of this type could in principle serve as a decision-support layer in diagnostic pipelines: predictions above a confidence threshold forwarded automatically, uncertain cases routed for specialist review. Based on the test-set confidence distribution of UQ-Mamba, a threshold of p>0.95 would auto-approve approximately 60–70% of cases while flagging the remainder. These figures are retrospective simulations on held-out benchmark data and must not be interpreted as deployment recommendations or clinical performance guarantees. Prospective validation through reader studies and decision-support trials with clinician involvement is required before any real-world application can be considered.

**Computational profile.** With 466K parameters and inference-only weights of approximately 1.8 MB, UQ-Mamba imposes low computational demands compared to ResNet-50 in the evaluated hardware and dataset configuration. Whether this efficiency advantage holds across heterogeneous device types, patient populations, and imaging protocols remains an open empirical question requiring multi-site validation.

**Interpretation of uncertainty components.** UQ-Mamba produces distinct epistemic and aleatoric uncertainty estimates derived via linearized error propagation. Conceptually, epistemic uncertainty reflects reducible model ignorance that may indicate a need for additional training data, while aleatoric uncertainty reflects irreducible noise in the input image—such as low resolution or acquisition artifacts—that may warrant repeat imaging. In practice, however, we have not empirically validated whether these two components behave as theoretically expected under controlled conditions. This remains an important limitation, discussed further below.

**Practical scope of the uncertainty signal.** As detailed in Section 4.3.3, UQ-Mamba’s primary triage value in its current form lies in its well-calibrated softmax confidence scores rather than the learned log-variance head. The log-variance signal is statistically non-trivial (Mann–Whitney p<0.001), but its ranking utility is subsumed by softmax confidence due to the shared-representation design. We acknowledge this as a significant practical limitation: the uncertainty decomposition into epistemic and aleatoric components currently provides calibration regularization during training but does not yield independent predictive information at inference time beyond what softmax confidence already encodes. To provide orthogonal triage value, the uncertainty and classification heads must be decoupled via auxiliary objectives or separate feature pathways. A further structural issue, independent of representation sharing, is that $\sigma_{\mathrm{epi},l}^{2}$ in Equation (10) is computed using $x_{\mathrm{mean},l}$—a single scalar summary of the block input—rather than a sample-specific high-dimensional feature. This means the epistemic uncertainty estimate is effectively a global input-magnitude scalar at each block, and its ability to produce truly sample-specific uncertainty estimates is limited even if the representation is decoupled. Addressing this would require computing the full Jacobian-based gradient, trading computational efficiency for richer uncertainty estimates. Quantifying the correlation between the log-variance signal and softmax confidence, computing AURC over the full coverage range, and evaluating out-of-distribution detection capability are identified as concrete next steps required to establish the practical utility of the uncertainty decomposition.

### 5.2. Calibration Analysis

The calibration advantage of UQ-Mamba over CNN baselines is a notable finding. ResNet-50 achieves marginally higher accuracy (90.14% vs. 89.71%) but exhibits an ECE of 0.0713, indicating that its confidence scores systematically overestimate correctness. **Training objective confound.** A direct ECE comparison between UQ-Mamba and CNN baselines is complicated by a difference in training objectives: UQ-Mamba minimizes a combined NLL + CE loss (Equation (11)), where the NLL term directly penalizes miscalibration, whereas ResNet-50 and EfficientNet minimize standard cross-entropy without any calibration objective. This difference systematically favors UQ-Mamba in ECE terms independently of any architectural advantage. The ECE gap (0.0713 vs. 0.0217) should therefore be interpreted with this confound in mind; isolating the architectural contribution would require training all baselines with the same NLL-augmented objective, which is deferred to future work. In any threshold-based system, such miscalibration would result in high-confidence incorrect predictions being passed without human review; however, whether this property would translate into clinical benefit requires prospective evaluation beyond these benchmarks.

The difference in calibration quality between OrganMNIST (ECE = 0.0217) and PathMNIST (ECE = 0.1188) is better attributed to dataset characteristics than to architectural properties. CT organ classification at 28×28 resolution is a structurally clean task with well-separated class boundaries, whereas colorectal histopathology involves texture-based discrimination among classes with substantial histological overlap. Temperature scaling reduces PathMNIST ECE by 10.1%, suggesting that the raw predictions are systematically but only moderately overconfident—a pattern amenable to post hoc correction.

### 5.3. Computational Efficiency

UQ-Mamba’s parameter count of 466K represents a roughly 50× reduction relative to ResNet-50, with inference latency of 2–3 ms and a training time of approximately 60 min on an Apple M-series system (MPS backend) for the OrganMNIST dataset. These figures reflect measurements with a specific hardware and dataset configuration and should not be construed as general performance guarantees. They do, however, indicate that the architecture does not impose prohibitive computational demands, which is relevant for deployment scenarios without access to dedicated GPU infrastructure.

**Portable and point-of-care imaging.** These efficiency characteristics are directly relevant to portable and point-of-care medical imaging deployments. Khalid et al. [29] document the expanding clinical role of portable CT scanners in intensive care units, operating theaters, and mobile stroke units, where on-device inference eliminates the latency and connectivity overhead of cloud-based processing. A model with 1.8MB inference-only weights and sub-3ms per-image latency on consumer hardware is compatible with the resource envelope of such devices. The combination of parameter efficiency, calibrated uncertainty estimation, and single-pass inference makes UQ-Mamba a candidate for evaluation in edge deployment scenarios, including portable dermoscopy devices and bedside diagnostic platforms in low-resource clinical settings. Whether the calibration and accuracy advantages demonstrated on standard benchmarks transfer to images acquired by portable devices—which may exhibit different noise profiles, acquisition geometries, and reconstruction artifacts—remains an open empirical question requiring dedicated validation studies.

### 5.4. Limitations

This study has several limitations that qualify the generalizability of the reported findings.

**Dataset scale and resolution.** Two of the four evaluated datasets (OrganMNIST and PathMNIST) are MedMNIST benchmarks at 28×28 resolution. While these benchmarks are widely used for methods-oriented research and enable reproducible comparison, they do not reflect the full range of challenges encountered in clinical imaging: high-resolution modalities (512×512 chest radiographs, gigapixel whole-slide images), severe class imbalance, label noise, and multi-site acquisition variability are not represented. The third dataset (HAM10000) uses dermoscopy images at 64×64 resolution and introduces real-world class imbalance; however, it is evaluated on a balanced three-class subset and does not represent the full diagnostic complexity of clinical dermatology. Validation on larger and more representative benchmarks—such as full-resolution CheXpert (this study used a 50K subset at 224×224), MIMIC-CXR, CAMELYON16, and full-resolution whole-slide image collections—is a necessary direction for future work before any claim of clinical generalizability can be made.

**Nature of the uncertainty propagation mechanism.** The uncertainty estimates produced by UQ-Mamba are derived via first-order (delta method) linearized error propagation on the learnable log-variance parameters of $\bar{A}$. This is a deterministic, closed-form computation—not a stochastic Bayesian posterior. As a result, the single-pass inference cost advantage over MC Dropout is genuine, but the epistemic/aleatoric decomposition is an approximation whose accuracy under conditions of high curvature or strong nonlinearity has not been established.

**Uncertainty validation.** Out-of-distribution detection experiments have been conducted (Section 4.3.4): epistemic uncertainty achieves strong OOD separation for dermoscopy (HAM10000 AUROC = 0.978) and moderate separation for histology (PathMNIST AUROC = 0.733). However, dataset shift analyses under controlled covariate shift (noise, blur, and contrast variation) and noisy-label robustness studies were not conducted and remain important directions. As the risk-coverage results (Section 4.3.3) and correlation analysis demonstrate, the aggregated log-variance uncertainty signal is less discriminative than softmax confidence as a case-ranking criterion ($r_{\mathrm{epi}-\mathrm{error}}=+0.010$, p=0.187; $r_{\mathrm{conf}-\mathrm{error}}=-0.534$, p<0.001). This gap between the theoretical properties of the epistemic/aleatoric decomposition and its empirical utility in selective prediction is an important open problem: the current formulation derives both the classification logits and the log-variance from the same pooled representation, which likely causes the two outputs to carry correlated rather than complementary information. Future work should explore auxiliary loss terms or architectural separations that encourage the uncertainty head to capture prediction error independently of softmax confidence.

**Epistemic and aleatoric decomposition.** The two uncertainty components are not validated under controlled experimental conditions. Confirming that epistemic uncertainty decreases with training set size and that aleatoric uncertainty increases with label noise would require dedicated ablation experiments beyond the scope of the present study and remains an important open direction.

**Aleatoric head collapse.** A notable failure mode was observed: the aleatoric uncertainty head consistently produces a near-constant output ($\sigma_{\mathrm{ale}}\approx 0.668$) regardless of input, yielding Pearson correlation undefined with any other signal (zero variance) and chance-level AUROC (0.500) in OOD detection. This collapse is attributed to a combination of the log-variance parameterization and the shared pooled representation: the NLL loss minimization drives the aleatoric head to a fixed-point equilibrium that minimizes the combined training objective without differentiating across samples. This renders the aleatoric component practically non-informative in the current formulation. Addressing this would require decoupled feature pathways, input-specific loss weighting, or auxiliary supervision (e.g., label-noise injection) to create a meaningful learning signal for the aleatoric head.

**Architectural scope.** The current architecture processes image patches as flat 1D sequences, without hierarchical or multi-scale feature extraction. Extension to multi-scale representations or volumetric inputs would be necessary to address three-dimensional imaging tasks and further improve performance on high-resolution data such as the 224×224 CheXpert radiographs evaluated here.

**Clinical validation.** All deployment scenarios described in this paper are simulations derived from test-set statistics. Prospective validation through reader studies or decision-support trials with clinician involvement is required before any real-world application. The efficiency and calibration results reported here should not be interpreted as evidence of clinical readiness.

**Calibration under distribution shift conditions.** It remains unknown whether the calibration observed on the MedMNIST test sets generalizes under covariate shift conditions arising from differences in imaging equipment, staining protocols, or patient demographics. Establishing this would require multi-site validation studies.

**Thermal and non-standard imaging modalities.** This study evaluates UQ-Mamba exclusively on four conventional radiological modalities: CT, histopathology, dermoscopy, and chest radiography. Thermal infrared imaging has been explored for applications including fever screening, peripheral vascular assessment, and breast thermography [30] but was not considered in this work. The behavior of the linearized uncertainty propagation mechanism on thermal image data—which exhibits distinct noise statistics and dynamic range characteristics relative to standard radiological modalities—remains an open question and represents a direction for future work.

## 6. Conclusions

This paper introduced UQ-Mamba, an SSM-based architecture that integrates uncertainty quantification into the model’s forward pass as a structural component rather than a post hoc correction. Uncertainty is propagated via linearized error propagation on the state transition matrix $\bar{A}$, producing approximate epistemic and aleatoric estimates in a single deterministic forward pass at 9.5% overhead over the deterministic baseline. Across four distinct medical imaging modalities—CT organ classification (OrganMNIST), colorectal histopathology (PathMNIST), dermoscopy lesion classification (HAM10000), and chest radiography (CheXpert)—UQ-Mamba consistently provides competitive accuracy with substantially improved calibration relative to larger CNN baselines. On OrganMNIST, the model achieves 89.71% test accuracy with an ECE of 0.0217 using only 466K parameters. On PathMNIST, it improves over the Mamba baseline by 2.42 percentage points with fair calibration following temperature scaling. On HAM10000 dermoscopy, it achieves 68.88% test accuracy (matching EfficientNet-B0 with 9× fewer parameters) with ECE = 0.0597—Good calibration on a third, visually distinct RGB modality, substantially better than ResNet-18 (ECE = 0.1189). On CheXpert chest radiographs (50K training subset), it achieves a mean AUC of 0.8196 across five competition labels on the official validation set (202 frontal views; CheXpert test-set labels are not publicly available), providing a proof-of-concept indication that the architecture extends to multi-label classification on real-world clinical imaging data; whether this constitutes generalization in a statistically meaningful sense requires evaluation in a controlled comparison against baselines trained on the identical 50K subset.

This work is presented as a proof-of-concept demonstrating that first-order error propagation through the SSM state transition matrix is a feasible and computationally efficient mechanism for producing calibrated predictions on standard benchmarks. The central contribution is not a claim of clinical readiness—the limitations outlined above, including single-seed evaluation, limited baseline coverage, and absence of clinical validation, make clear that substantial further work remains. Rather, the core argument is that propagating uncertainty through the SSM’s state transition matrix via closed-form first-order approximation provides a structurally motivated, parameter-efficient mechanism for calibration. The empirical results support the feasibility of this design choice across four distinct medical imaging tasks and modalities. Importantly, the triage utility of the architecture in its current form rests primarily on its well-calibrated softmax confidence scores; the learned log-variance signal requires architectural decoupling from the classification head before it can provide independent diagnostic value.

Future work should prioritize validation on higher-resolution datasets and more clinically representative benchmarks, controlled ablation experiments to verify the epistemic and aleatoric decomposition under distributional perturbations (varying training set size and label noise levels), out-of-distribution robustness evaluation to assess the reliability of the uncertainty estimates beyond the test distributions examined here, and architectural modifications to decouple the uncertainty and classification heads so that the log-variance signal provides triage information complementary to—rather than redundant with—softmax confidence.

## Figures and Tables

**Table 1:** Summary of datasets used in this study.

Dataset	Modality	Classes	Train/Val/Test	Resolution	Access
OrganMNIST [25]	CT (grayscale)	11	34,561/6491/17,778	28×28	Public
PathMNIST [25]	Histopathology	9	89,996/10,004/7180	28×28×3	Public
HAM10000 * [26]	Dermoscopy	3	≈2400/≈300/≈300	64×64×3	Public
CheXpert [27]	Chest X-ray	5 (multi-label)	50,000/202/– ^†^	224×224	Restricted

* Balanced 3-class subset (1000 images per class: nv, mel, bkl) of the full 10,015-image HAM10000 collection. ^†^ CheXpert test-set labels are not publicly available; evaluation uses the official validation set (202 frontal images). CheXpert training used a 50K random subset of the full 191K frontal images.

**Table 2:** UQ-Mamba configuration.

Component	Parameter	Value
Input	Image Size	28×28×C (Organ: C=1; Path: C=3);
		64×64×3(HAM); 224×224×1 (CheXpert)
	Patch Size	2 (Organ), 4 (Path/HAM), 16 (CheXpert)
	Number of Patches	196 (Organ/CheXpert), 49 (Path), 256 (HAM)
Backbone	Embedding Dim (*d*)	128
	SSM State Dim (*N*)	16
	Depth (*L*)	6 blocks
	Dropout	0.1
Training	Optimizer	AdamW, lr = $10^{-3}$
	Batch Size	256 (Organ), 128 (Path), 32 (HAM/CheXpert)
	Epochs	30 (Organ/Path/HAM), 20 (CheXpert)
	Loss Weight (λ)	0.2
Efficiency	Total Parameters	**466K**
	Checkpoint Size (train)	35 MB *
	Inference Weights	1.8 MB
	Inference Time	2.3 ms

* Training checkpoint includes model weights plus AdamW optimizer state (two moment buffers). Inference-only weights are 466K×4 bytes = 1.8 MB. Bold indicates the proposed UQ-Mamba model.

**Table 3:** Efficiency comparison on OrganMNIST. CNN baselines: ResNet [7], EfficientNet [8].

Model	Params	Size (MB)	Train (min)	Infer (ms)	FLOPs (M)
ResNet-50	23.5M	90	240	8.2	412 ^†^
ResNet-18	11.2M	43	180	6.5	289 ^†^
EfficientNet-B0	4.01M	16	150	5.8	198 ^†^
**UQ-Mamba**	**0.466M**	**1.8**	**60**	**2.3**	**87**
*vs. ResNet-50*	50×	50×	4×	3.6×	4.7×

Bold indicates the proposed UQ-Mamba model. Size (MB): inference-only weights for all models. ^†^ FLOPs for CNN baselines are measured at 28 × 28 input resolution (the actual OrganMNIST evaluation size) and therefore differ from canonical values typically quoted for 224 × 224 inputs (e.g., ResNet-50 at 224 × 224 requires ∼4 GFLOPs). UQ-Mamba FLOPs are measured at 28 × 28 with 196 tokens.

**Table 4:** Inference overhead by UQ method. MC Dropout from Gal & Ghahramani [10]; Deep Ensemble from Lakshminarayanan et al. [11].

UQ Method	Fwd. Passes	Infer. Time	Rel. Cost
Baseline (no UQ)	1	2.1 ms	1.0×
**UQ-Mamba (ours)**	**1**	**2.3** **ms**	**1.1×**
MC Dropout (*T* = 30)	30	63 ms	30×
Deep Ensemble (*N* = 5) ^‡^	5	10.5 ms	5×
Temperature Scaling	1	2.2 ms	1.05×

Bold indicates the proposed UQ-Mamba model. ^‡^ Deep ensembles aggregate predictions from independently trained models and improve calibration but do not produce structured epistemic/aleatoric decomposition. Included for inference-cost reference only. All inference times were measured on the same Apple M-series MPS backend (single image, batch size 1, averaged over 100 runs after 10 warm-up iterations, single-thread, PyTorch 2.1, float32 precision). MC Dropout and Deep Ensemble times are empirical measurements on this hardware, not theoretical estimates. The MPS backend may not fully exploit the parallel acceleration advantage of CNN-based methods available on dedicated GPU hardware; the relative cost figures should therefore be interpreted as hardware-specific rather than architecture-general.

**Table 5:** OrganMNIST test set results. ResNet baselines from He et al. [7]; EfficientNet from Tan & Le [8]; MC-Dropout from Gal & Ghahramani [10].

Model	Params	Test Acc (%)	ECE ↓	Brier ↓	UQ
ResNet-50	23.5M	90.14	0.0713	–	None
ResNet-18	11.2M	89.10	0.0642	–	None
EfficientNet-B0	4.01M	89.40	0.0687	–	None
ResNet-18+MCD	11.2M	90.54	0.0611	0.1540	MCD ^*a*^
EffNet-B0+MCD	4.01M	89.71	0.0650	0.1651	MCD ^*a*^
Baseline Mamba	0.466M	88.36	– ^*b*^	–	None
**UQ-Mamba**	**0.466M**	**89.71**	**0.0217**	**0.1523**	**1-pass**

Bold indicates the proposed UQ-Mamba model. ^*a*^ MC-Dropout, *T* = 30 forward passes. Brier scores are not reported for deterministic CNN baselines because their softmax outputs were not retained with per-sample logging during evaluation; they are reported for MC-Dropout variants where probability arrays were collected. ^*b*^ ECE is not reported for the deterministic Baseline Mamba for the same reason; ECE for ResNet and EfficientNet was computed as these baselines were evaluated with probability logging enabled.

**Table 6:** PathMNIST test set results. ResNet baselines from He et al. [7]; EfficientNet from Tan & Le [8]; MC-Dropout from Gal & Ghahramani [10]. ECE is not reported for deterministic models (no uncertainty quantification).

Model	Test Acc (%)	ECE ↓	Brier ↓	UQ Method
ResNet-18	74.42	– ^*b*^	–	None
ResNet-18 + MC-Dropout	74.42	0.1280	0.3911	MC-Dropout (*T* = 30)
EfficientNet-B0	74.21	– ^*b*^	–	None
EfficientNet-B0 + MC-Dropout	74.21	0.0395	0.3746	MC-Dropout (*T* = 30)
Baseline Mamba	75.17	– ^*b*^	–	None
**UQ-Mamba (Ours)**	**77.59**	**0.1188**	**0.3481**	**Single-pass**

Bold indicates the proposed UQ-Mamba model. ^*b*^ ECE not reported for deterministic models (ResNet, EfficientNet, Baseline Mamba) because their per-sample softmax probability arrays were not retained during evaluation; ECE for MC-Dropout variants was computed from the collected probability arrays. Deterministic CNN accuracy values match their MC-Dropout counterparts because the backbone weights are identical; MC-Dropout applies stochastic inference at test time without retraining. UQ-Mamba ECE = 0.1188 is reported after temperature scaling (*T* = 1.14); raw ECE before scaling is 0.1322. The “background” class in PathMNIST is included as part of the standard MedMNIST benchmark protocol and does not represent a clinically meaningful tissue category.

**Table 7:** Effect of temperature scaling on CNN baseline calibration. T>1: model was overconfident; T<1: underconfident. UQ-Mamba ECE included for reference (no temperature scaling applied).

Dataset	Model	ECE (raw)	ECE (TS)	*T*	ΔECE
HAM10000	ResNet-18	0.1189	0.0585	1.371	−0.0604
	EfficientNet-B0	0.0427	0.0643	0.837	+0.0216
	**UQ-Mamba (ours)**	**0.0597**	–	–	–
CheXpert	ResNet-18	0.1445	0.1286	0.963	−0.0159
	EfficientNet-B0	0.1375	0.1164	0.902	−0.0211
	**UQ-Mamba (ours)**	**0.0890**	–	–	–

Bold indicates the proposed UQ-Mamba model. Temperature *T* optimized on the validation set by minimizing NLL (HAM10000) or mean binary cross-entropy (CheXpert). EfficientNet-B0 HAM10000 ECE increases under TS because the model was already well-calibrated; T<1 pushes confidence higher, amplifying the existing mild underconfidence.

**Table 8:** Selective prediction evaluation. AURC and E-AURC are lower-is-better; AUROC and Coverage@Risk are higher-is-better. UQ-Mamba (conf) uses softmax confidence; epistemic/aleatoric/total use the learned uncertainty head components.

Dataset	Model / Score	AURC ↓	E-AURC ↓	AUROC ↑	Cov@R10% ↑
HAM10000	ResNet-18 (conf)	0.1957	0.1260	0.7104	0.238
	EfficientNet-B0 (conf)	0.1565	0.0993	0.7371	0.350
	UQ-Mamba (conf)	**0.1667**	**0.1120**	**0.7144**	**0.311**
	UQ-Mamba (epistemic)	0.3695	0.3148	0.3743	0.003
	UQ-Mamba (aleatoric)	0.2679	0.2132	0.5000	0.034
CheXpert	ResNet-18 (conf)	0.3294	0.0953	0.8547	0.198
	EfficientNet-B0 (conf)	0.3193	0.0897	0.8526	0.218
	UQ-Mamba (conf)	**0.3795**	**0.1171**	**0.8300**	**0.035**
	UQ-Mamba (epistemic)	0.6846	0.4222	0.4395	0.000
	UQ-Mamba (aleatoric)	0.7241	0.4617	0.5000	0.000

Bold indicates UQ-Mamba (conf) rows (proposed model). AURC oracle is the risk-coverage area achievable by a perfectly calibrated oracle; E-AURC subtracts this from the empirical AURC to quantify excess abstention cost. CheXpert error is defined as any incorrect binary prediction across the five competition labels. Coverage@R10% = fraction of samples retained when error rate is capped at 10%.

**Table 9:** Out-of-distribution detection AUROC. ID dataset: OrganMNIST test set (n=17,778). Higher is better.

OOD Dataset	Epistemic	Aleatoric	Total	1 − MaxProb
PathMNIST	0.7327	0.500 ^*a*^	0.7327	0.8809
HAM10000	0.9776	0.500 ^*a*^	0.9776	0.7763

^*a*^ Aleatoric uncertainty collapsed to a constant ≈0.668 across all inputs (see Section 5.4), yielding chance-level AUROC. Epistemic AUROC computed as $P({\mathrm{score}}_{\mathrm{OOD}}>{\mathrm{score}}_{\mathrm{ID}})$. 1 − MaxProb uses softmax confidence as the ID signal.

**Table 11:** HAM10000 test set results (balanced 3-class: nv/mel/bkl, 1000 samples per class). Val accuracy reflects the best checkpoint; test accuracy is evaluated on the held-out lesion-split test set. All models were trained under the same protocol (30 epochs, cosine annealing, and lesion-level split) for controlled comparison.

Model	Params	Val Acc (%)	Test Acc (%)	ECE ↓	UQ
ResNet-18	11.2M	75.33	65.14	0.1189	None
EfficientNet-B0	4.01M	76.00	68.20	0.0427	None
**UQ-Mamba (Ours)**	**0.466M**	**75.00**	**68.88**	**0.0597**	**1-pass**

Bold indicates the proposed UQ-Mamba model. ECE computed over 15 bins on the held-out test set. Epistemic = 1.4944; Aleatoric = 3.2498 (batch-averaged scalars for UQ-Mamba). All three models were trained with identical lesion-level splits, protocols, and hardware. UQ-Mamba achieves the highest test accuracy with 24× fewer parameters than ResNet-18 and provides native uncertainty decomposition unavailable in CNN baselines.

**Table 12:** CheXpert validation set results (frontal views, five competition labels, and 50K training subset). AUC-ROC is reported per class and as mean (mAUC). All models were trained on the same 50K subset under the same protocol for controlled comparison.

Model	Params	Atel.	Card.	Cons.	Edema	Pl.Eff.	mAUC
ResNet-18	11.2M	0.7746	0.8675	0.8846	0.8811	0.8942	0.8604
EfficientNet-B0	4.01M	0.7925	0.8539	0.8912	0.8987	0.9130	0.8699
**UQ-Mamba (Ours)**	**0.466M**	**0.7612**	**0.7493**	**0.8744**	**0.8656**	**0.8474**	**0.8196**

Bold indicates the proposed UQ-Mamba model. UQ-Mamba: ECE = 0.0890 (ResNet-18: 0.1445; EfficientNet-B0: 0.1375); Epistemic = 0.3161; Aleatoric = 0.1009. 50K of 191K available training images used. UQ-Mamba’s lower mAUC relative to CNN baselines is attributable to its 9–24× smaller parameter count; its ECE advantage (0.0890 vs. 0.14+) reflects better-calibrated predictions—an important property for clinical triage.

**Table 13:** Ablation study: all twelve configurations on OrganMNIST (validation accuracy). Each dimension is ablated independently with all other hyperparameters held at the selected (✔) values. Validation accuracy is inflated relative to test accuracy due to shared CT volumes (see Section 3.2.1).

Dimension	Value	Val Acc (%)	Selected
Depth (*L*)	2	94.91	✔
	6	97.98	
	8	97.72	
Patch Size (*P*)	2	97.98	✔
	4	96.22	
	8	94.11	
Embed Dim (*d*)	64	94.88	✔
	128	97.98	
	256	93.52	
SSM State (*N*)	8	97.55	✔
	16	97.98	
	32	97.67	

All values are OrganMNIST validation accuracy from single training runs (seed = 42). Configurations with “comparable” results (N = 8, N = 32) fall within 0.5 pp of the selected value. See Figure 6 for a visual comparison across all dimensions.

**Table 14:** UQ head contribution ablation on OrganMNIST. Both models use identical architecture and training procedure; only the SSM log-variance parameters differ.

Model	Test Acc (%)	ECE ↓	Epistemic	Aleatoric
Mamba + NLL only	89.64	0.0307	7.3817	–
**UQ-Mamba (full)**	**89.71**	**0.0301**	**0.8489**	**0.6585**
Δ (full − NLL-only)	+0.07	−0.0006		

Bold indicates the proposed UQ-Mamba (full) model.

**Table 15:** Extended UQ ablation on OrganMNIST (30 epochs, seed = 42). All configurations use the full UQ-Mamba architecture; only the active uncertainty pathways and UQ head capacity differ. NLL: negative log-likelihood loss term; SSM: state space model log-variance propagation; CE: plain cross-entropy.

Configuration	Test Acc (%)	ECE ↓	Epistemic	Aleatoric
No UQ (CE only)	**89.70**	0.0492	≫1000	5.00
NLL only (no SSM prop.)	84.41	0.0128	0.1297	5.00
Epistemic only	85.65	0.0183	0.0738	5.00
Aleatoric only	84.40	**0.0057**	0.0796	0.8300
Full UQ ($d_{s}$ = 16)	85.84	0.0198	0.0412	0.7346
Full UQ ($d_{s}$ = 8)	85.12	0.0180	0.0487	0.8484

Bold indicates the best value per column. No UQ uses plain cross-entropy; all other configurations use NLL. The large epistemic value for No UQ reflects uninitialized log-variance parameters at exp(0). Aleatoric only achieves the lowest ECE (0.0057) but at 5.3 pp lower accuracy than No UQ, suggesting a trade-off between uncertainty calibration and discriminative performance with the NLL objective. Full UQ with $d_{s}=16$ offers the best accuracy among UQ configurations with interpretable uncertainty decomposition.

**Table 16:** Multi-seed reproducibility on OrganMNIST (patch_size = 2, depth = 6). Three independent training runs with different random seeds.

Seed	Test Acc (%)	ECE ↓	Epistemic	Aleatoric
42	89.76	0.0304	0.8710	0.6683
123	89.40	0.0325	1.0293	0.6904
789	87.16	0.0103	0.0663	1.3668
**Mean** ± **Std**	88.77±1.41	0.0244±0.0122	–	–

All runs use identical architecture, training protocol, and hardware; only the random seed differs. ECE computed over 15 bins on the held-out test set. Std is the sample standard deviation across three runs.

## Data Availability

The MedMNIST datasets (OrganMNIST and PathMNIST) are publicly available at https://medmnist.com/. HAM10000 is available at the Harvard Dataverse https://doi.org/10.7910/DVN/DBW86T. CheXpert is available from Stanford ML Group upon registration (https://stanfordmlgroup.github.io/competitions/chexpert/ (accessed on 20 June 2026)). Training code, hyperparameter configurations, and model checkpoints sufficient to reproduce all reported results will be made publicly available via a GitHub repository, available at https://github.com/aligunesgit/uq-mamba-medical-imaging.

## Abbreviations

The following abbreviations are used in this manuscript:

AI	Artificial intelligence
AUC	Area under the ROC curve
CNN	Convolutional neural network
CT	Computed tomography
ECE	Expected calibration error
MC	Monte Carlo
MCD	Monte Carlo Dropout
MPS	Metal Performance Shaders
NLL	Negative log-likelihood
ROC	Receiver operating characteristic
SSM	State space model
UQ	Uncertainty quantification
